# Genome-Wide Identification and Characterization of Drought Stress Responsive microRNAs in Tibetan Wild Barley

**DOI:** 10.3390/ijms21082795

**Published:** 2020-04-17

**Authors:** Cheng-Wei Qiu, Li Liu, Xue Feng, Peng-Fei Hao, Xiaoyan He, Fangbin Cao, Feibo Wu

**Affiliations:** 1 Institute of Crop Science, Department of Agronomy, College of Agriculture and Biotechnology, Zijingang Campus, Zhejiang University, Hangzhou 310058, China; 3130100260@zju.edu.cn (C.-W.Q.); 21316026@zju.edu.cn (X.F.); 11816004@zju.edu.cn (P.-F.H.); hexiaoyan@zju.edu.cn (X.H.); 2 Department of Applied Engineering, Zhejiang Economic and Trade Polytechnic, Hangzhou 310018, China; liul@zjiet.edu.cn; 3 Jiangsu Co-Innovation Center for Modern Production Technology of Grain Crops, Yangzhou University, Yangzhou 225009, China

**Keywords:** *Hordeum vulgare* L. ssp. *spontaneum*, dehydration stress, next-generation sequencing, miRNA, target genes

## Abstract

Drought stress is a major obstacle to agricultural production. Tibetan wild barley with rich genetic diversity is useful for drought-tolerant improvement of cereals. MicroRNAs (miRNAs) play critical roles in controlling gene expression in response to various environment perturbations in plants. However, the genome-wide expression profiles of miRNAs and their targets in response to drought stress are largely unknown in wild barley. In this study, a polyethylene glycol (PEG) induced drought stress hydroponic experiment was performed, and the expression profiles of miRNAs from the roots of two contrasting Tibetan wild barley genotypes XZ5 (drought-tolerant) and XZ54 (drought-sensitive), and one cultivated barley Tadmor (drought-tolerant) generated by high-throughput sequencing were compared. There were 69 conserved miRNAs and 1574 novel miRNAs in the dataset of three genotypes under control and drought conditions. Among them, seven conserved miRNAs and 36 novel miRNAs showed significantly genotype-specific expression patterns in response to drought stress. And 12 miRNAs were further regarded as drought tolerant associated miRNAs in XZ5, which mostly participate in gene expression, metabolism, signaling and transportation, suggesting that they and their target genes play important roles in plant drought tolerance. This is the first comparation study on the miRNA transcriptome in the roots of two Tibetan wild barley genotypes differing in drought tolerance and one drought tolerant cultivar in response to PEG treatment. Further results revealed the candidate drought tolerant miRNAs and target genes in the miRNA regulation mechanism in wild barley under drought stress. Our findings provide valuable understandings for the functional characterization of miRNAs in drought tolerance.

## 1. Introduction

Drought, one of the most widespread natural disasters in the world, seriously affects agricultural production [1]. In recent years, prolonged drought has caused severe damage to crops in various agricultural areas worldwide [2]. Breeding for drought tolerant crop cultivars is one of the most effective approaches to reduce the impact of dehydration on plant growth and achieve greater economic gains. During evolution, plants gradually developed robust mechanisms (morphological, physiological, biochemical and molecular) to adapt to drought stress [3]. In general, there are different drought response patterns in crop varieties, and any two varieties with similar drought tolerance may exhibit different gene expression and metabolism pathways. Therefore, it is important to identify drought tolerant genetic resources and to understand the specific mechanism, to prevent reduced crop production.

MicroRNAs (miRNAs) are well-studied species of small noncoding RNAs [4]. In plants, miRNAs are considered important regulators to alter gene expression in many aspects, including cleavage of target messenger RNAs, translational repression, DNA methylation and the regulation of miRNA activities [5]. Large numbers of studies have demonstrated that miRNAs are involved in numerous biological and metabolic processes in plants, including, but not limited to, growth, development, hormone signaling and stress response [6,7]. Many miRNAs have already been identified in model plants under drought stresses, which mainly played important roles in ABA response, Auxin signaling, cell growth, osmotic regulation, antioxidant, photosynthesis and respiration [8,9,10,11]. In barley, different miRNA expression profile was observed in cultivated barley Golden Promise under drought condition via deep sequencing [12]. Ferdous et al. [13] also identified four miRNAs differentially accumulated in four barley cultivars, and further determined that *Hv*-miR827 was able to enhance drought tolerance of barley [14]. Similarly, Fard et al. [15] compared the differential miRNA expression pattern of two barley cultivars, showing different performance under drought stress. Zhou et al. [16] found that a *Triticeae*-specific miRNA hvu-miRX targeted various genes and conferred drought tolerance in barley. However, there is less information about the expression profiles of miRNAs and their target genes in the roots of wild barley genotypes differing in drought tolerance.

Barley (*Hordeum vulgare* L.), one of the most important cereal crops, is extensively used as the raw material for malting and animal feed. Because of its relatively high drought tolerance, barley has been often considered as a model plant to identify the underlying mechanism of drought tolerance in cereal crops. However, cultivated barley has lost its genetic diversity during the domestication process, making it hypersensitive to both abiotic and biotic stress. On the contrary, the Tibetan wild barley, one of the progenitors of cultivated barley, has developed effective mechanisms in response to environmental perturbations under the harsh conditions of the Qinghai-Tibet Plateau in China, and also exhibits rich genetic diversity on the trait of drought tolerance [17,18]. In our previous studies, we demonstrated that the Tibetan wild barley genotype XZ5 showed higher drought tolerance compared to globally accepted drought tolerant *cv*. Tadmor [19]. Thus, it is imperative to investigate the special mechanisms in drought tolerance of wild barley. 

Drought tolerance responses in crops have been well documented at the physiological, biochemical and transcriptional levels, but miRNA-mediated regulation is still poorly understood, especially in wild barley. Consequently, investigating the miRNA transcriptome in wild barley using high-throughput sequencing technology could be an effective approach for determining drought tolerance associated miRNAs and their target genes, which could be beneficial to the breeding of cultivars with improved drought tolerant. In the present study, a comparative analysis was performed on the miRNA transcriptomes generated by next-generation sequencing of the two Tibetan wild barley accessions XZ5 (drought-tolerant) and XZ54 (drought-sensitive), and one cultivated barley Tadmor (drought-tolerant) in the root under control and drought conditions. As a result, 12 miRNAs were identified to be significantly down-regulated in XZ5, but unchanged or up-regulated in the XZ54 and Tadmor under drought stress; thus, they were considered the most relevant group associated with drought tolerance. The functional analysis of miRNAs and their targets showed these miRNA–mRNA pairs were involved in regulation of gene expression, metabolism, signaling and transportation. These findings provided new insights in the understanding the role of barley miRNAs in drought tolerance.

## 2. Results

### 2.1. High-Throughput Sequencing of Barley Small RNAs

A total of 27,450,467, 27,644,000, 28,370,268, 26,932,023, 27,800,513 and 28,370,268 raw reads with 18–30 nt in lengths were generated from six root libraries (Control: XZ5, XZ54, Tadmor; Drought: XZ5, XZ54, Tadmor), respectively (Table 1). After filtration, 24,339,157, 24,939,692, 26,245,145, 24,392,408, 25,771,155 and 25,550,026 clean reads were remained in these libraries. These clean data were mapped onto the genome of *Hordeum vulgare* L. and other sRNA databases using the Bowtie2 program. Consequently, 17,310,505 (71.12%), 18,034,873 (72.31%), 18,772,380 (71.53%) clean reads were matched to the reference genome in the control libraries of XZ5, XZ54 and Tadmor that included 1,655,750, 1,705,665, 2,127,598 miRNA reads, respectively. Similarly, 16,809,346 (68.91%), 19,179,027 (74.42%), 18,558,569 (72.64%) clean reads were matched in the drought libraries of XZ5, XZ54 and Tadmor, including 1,401,558, 1,312,933 and 1,553,489 miRNA reads, respectively. It was observed that only few reads were matched with the reported miRNAs in databases, while others were still unknown, which indicates the complexity of miRNAs in barley. 

**Figure 1:**
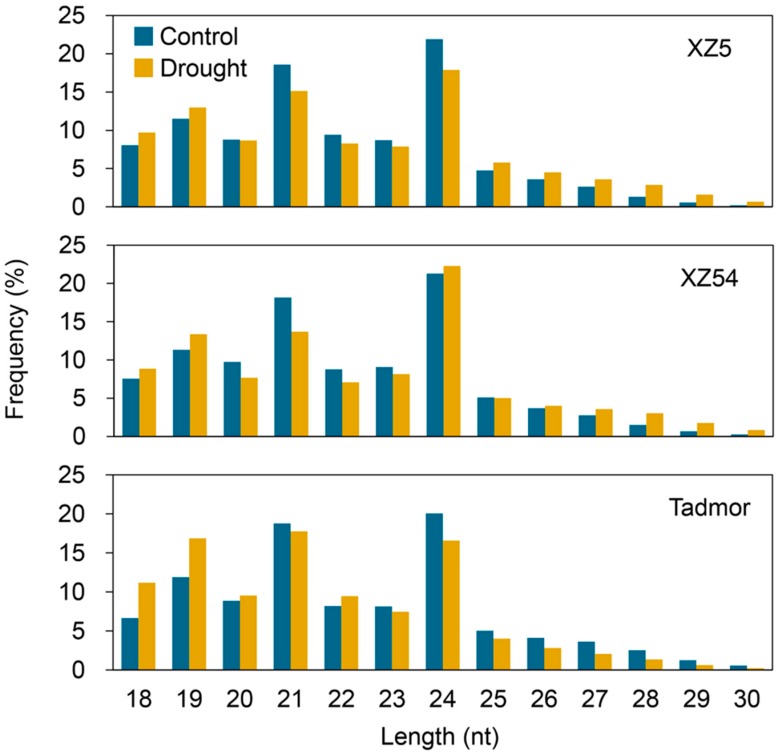
Length distribution of small RNAs in roots of XZ5, XZ54 and Tadmor after 24h 20% PEG-6000 treatment. The X-axis represents the small RNA length (nucleotide) and the Y-axis represents the percentage of small RNA reads. Control corresponds to hydroponically grown barley in basic nutrition solution and Drought represents 20% PEG-6000 treatment.

Based on length distribution, we found that small RNAs with length of 21 and 24 nt were abundant in our dataset, of which 24 nt is the most abundant length (Figure 1). There were similar length distributions of small RNAs among the three genotypes under control condition. However, the length distribution of small RNAs changed significantly under the drought condition, showing an increased number of 18, 19, 25, 26, 27, 28 nt small RNAs and fewer 21, 22, 23 nt small RNAs. Interestingly, the number of 24 nt small RNAs decreased in XZ5 and Tadmor, but increased in XZ54. These results suggested that the expression profiles of small RNAs was altered by drought stress in barley roots.

### 2.2. Identification of Conserved miRNAs and Novel miRNAs

**Figure 2:**
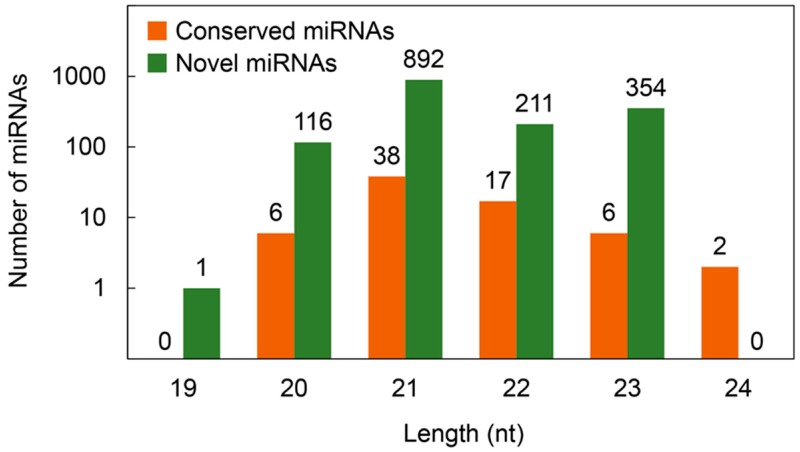
Length based distribution of conserved and novel microRNAs (miRNAs) in barley roots.

The conserved miRNAs were analyzed in our dataset using miRbase (Ver 21.0). Based on sequence similarity, a total of 69 conserved miRNAs were identified. Among them, 28 of them belonged to 13 miRNA families, and the remaining 41 were still undefined (Table S1). As shown in Figure 2, most of the 13 families had only one or two conserved miRNAs, with the largest family, MIR5067 containing six members. Among these 69 miRNAs, the highest abundant length was 21 nt, accounting for 55.07% of the total miRNAs, followed by 22 nt (24.64%) and 24 nt (2.90%, least abundant length).

To analyze the evolutionary relationship of miRNAs, the 13 conserved miRNA families of barley were compared with other species, such as *Triticum aestivum*, *Brachypodium distachyon*, *Aegilops tauschii*, *Oryza sativa* and *Zea mays* (Table S2). It was found that MIR156, MIR159 and MIR171_1 were conserved in these species, while MIR5048 was only identified in *Hordeum vulgare*, indicating MIR5048 might have special roles in barley different from other crops.

Novel miRNAs were predicted in barley using RNAfold and miReap softwares. A total of 1574 novel miRNAs were identified in our dataset (Table S3). Among them, 944, 972 and 1011 novel miRNAs were identified in XZ5, XZ54 and Tadmor under control condition, while 797, 877 and 965 novel miRNAs were identified in those under drought condition, respectively. These predicted novel miRNAs ranged from 19 to 23 nt in length, a peak was at 21 nt, including 892 miRNAs (Figure 2).

### 2.3. Genotypic Differences in miRNA Expression Profiles under Drought Stress in Barley

**Figure 3:**
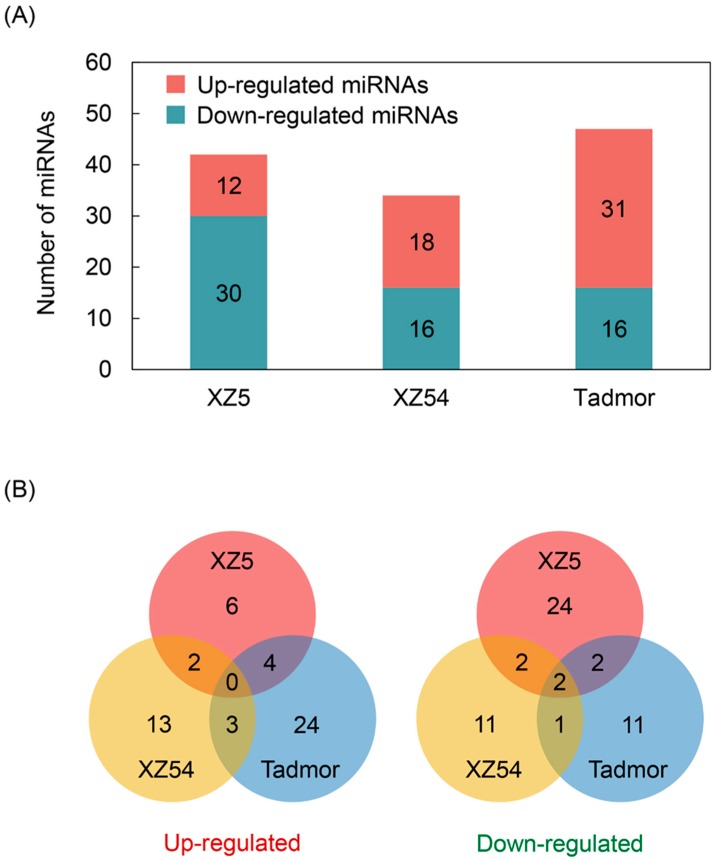
Root transcriptome profiles of drought stress-responsive miRNAs in three barley genotypes. (**A**) Up- and down-regulated miRNAs of XZ5, XZ54 and Tadmor in response to drought stress. (**B**) Venn diagram of up- and down-regulated miRNAs in three barley genotypes. Fold change (drought vs control) is log_2_N, log_2_N ≥ 1 are up-regulated, between 0 < |log_2_N| < 1 are unchanged and log_2_N ≤ −1 are down-regulated, *p*-values ≤ 0.01 and Q-value ≤ 0.001.

The expression profiles of miRNAs were normalized by Transcripts Per Million (TPM) for further comparative analysis (Table S4). Then, the drought stress responsive miRNAs in barley were identified by DEGs analysis software with a fold change of at least 1.0. It was found that the expression level of miRNAs in three barley genotypes were significantly altered after 24 h drought stress induced by 20% PEG-6000. Among the 69 conserved miRNAs and 1574 novel miRNAs, 31 conserved miRNAs and 115 novel miRNAs were significantly affected by drought stress with Q-value < 0.001 (Table S5). Specifically, under drought stress, there were 12, 41 and 30 miRNAs up-regulated, unchanged and down-regulated in XZ5, respectively; while 18, 48 and 16 miRNAs were up-regulated, unchanged and down-regulated in XZ54; and the 31, 53 and 16 miRNAs were up-regulated, unchanged and down-regulated, respectively, in Tadmor (Figure 3A and Table S6). The number of down-regulated miRNAs in XZ5 was greater than that in XZ54 and Tadmor, indicating that more target genes were up-regulated, which may contribute to enhanced drought tolerance in XZ5. In addition, it was found that Novel-m1509-3p and Novel-m2061-5p were significantly down-regulated in all three genotypes (Figure 3B).

**Figure 4:**
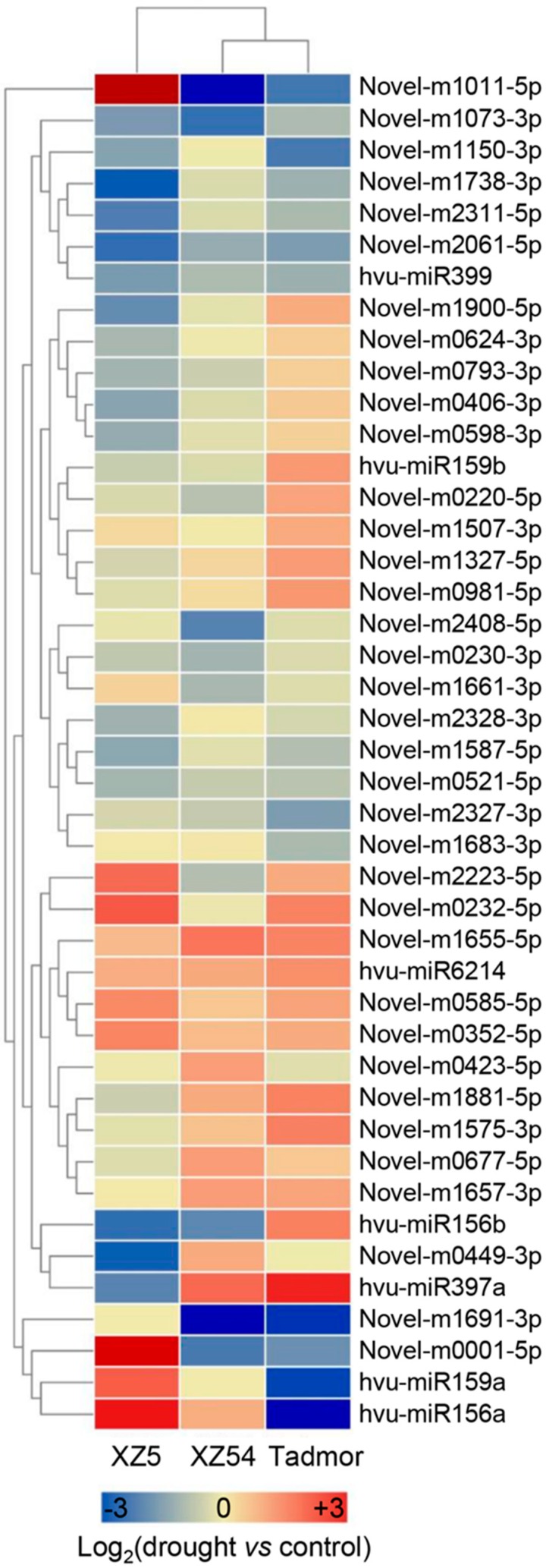
The hierarchical clustering analysis of drought-responsive miRNAs from roots of XZ5, XZ54 and Tadmor. Hierarchical clustering of differently expressed miRNAs was displayed by Euclidean distance and complete cluster methods as a measurement of similarity.

**Figure 5:**
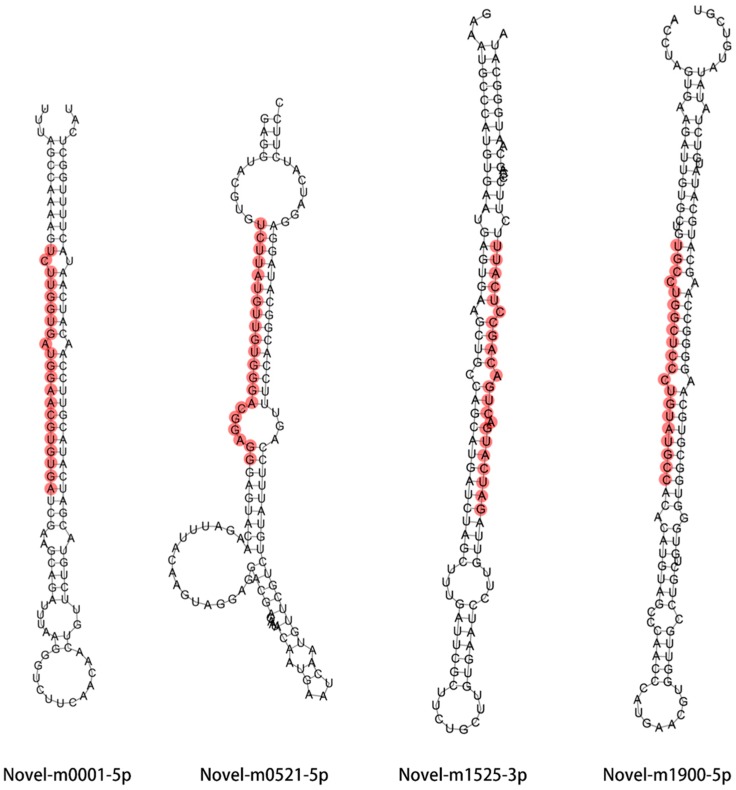
Secondary structure prediction of four novel miRNAs differently expressed in XZ5, XZ54 and Tadmor in response to drought stress. Red bars indicate the mature miRNAs.

A total of seven conserved miRNAs and 36 novel miRNAs were considered and determined as drought responsive miRNAs, according to their genotypic differences in miRNA expression profile against drought stress (Figure 4). MicroRNAs have been recognized as negative regulator of their target genes, so we mainly focus on drought responsive miRNAs that were down-regulated in XZ5, but slightly altered or up-regulated in XZ54 and Tadmor. Thus, two conserved and 10 novel miRNAs were considered as drought tolerant miRNAs in XZ5 and were further investigated (Table 2). In addition, the putative secondary structures of four drought tolerant associated novel miRNAs are shown in Figure 5.

### 2.4. qRT-PCR Validation for miRNA Expression

**Figure 6:**
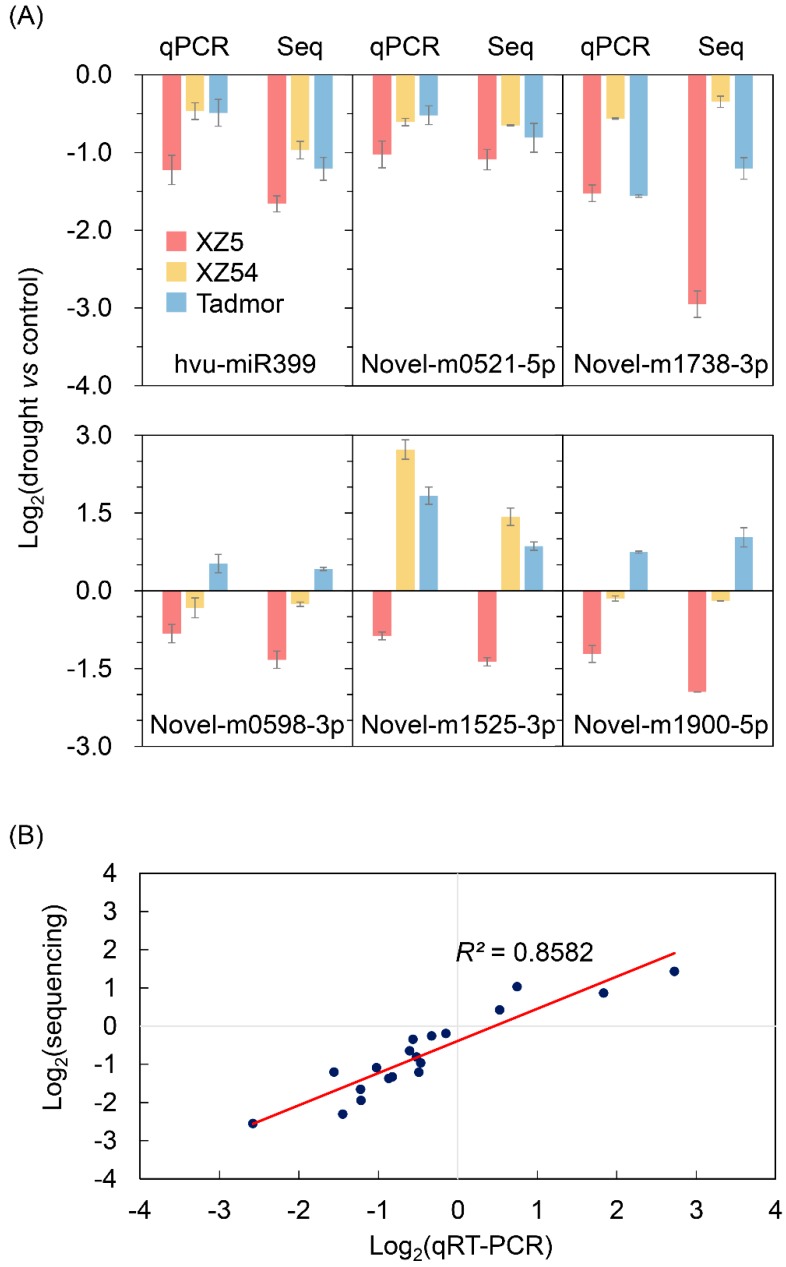
qRT-PCR confirmation of miRNAs identified in XZ5, XZ54 and Tadmor in response to drought stress. (**A**) Expression patterns of six mRNAs in the qRT-PCR and sequencing dataset. (**B**) Correlation analysis of quantitative real-time PCR and sequencing data of miRNAs.

The expression levels of several drought tolerant associated miRNAs were further determined and validated by quantitative real time PCR (qRT-PCR). As shown in Figure 6, the sequencing and qRT-PCR results were well fitted by linear regression, with a regression coefficient of 0.8582. As anticipated, the specific values of fold change from qRT-PCR were not completely identical to those from sequencing results, but both qRT-PCR and sequencing results exhibited similar trends on miRNA expression in response to drought stress. These results indicated that the results of qRT-PCR were consistent with the sequencing dataset.

### 2.5. Annotation and Functional Analysis of miRNA Target Genes

**Figure 7:**
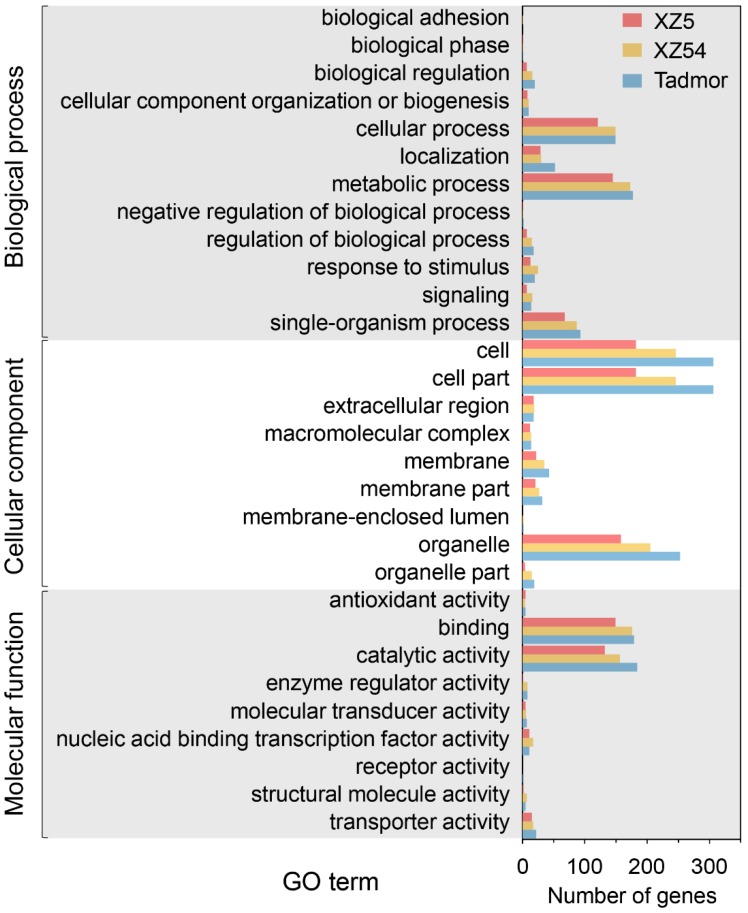
Gene Ontology (GO) analysis for target genes of differently expressed miRNA in response to drought stress in XZ5, XZ54 and Tadmor.

**Figure 8:**
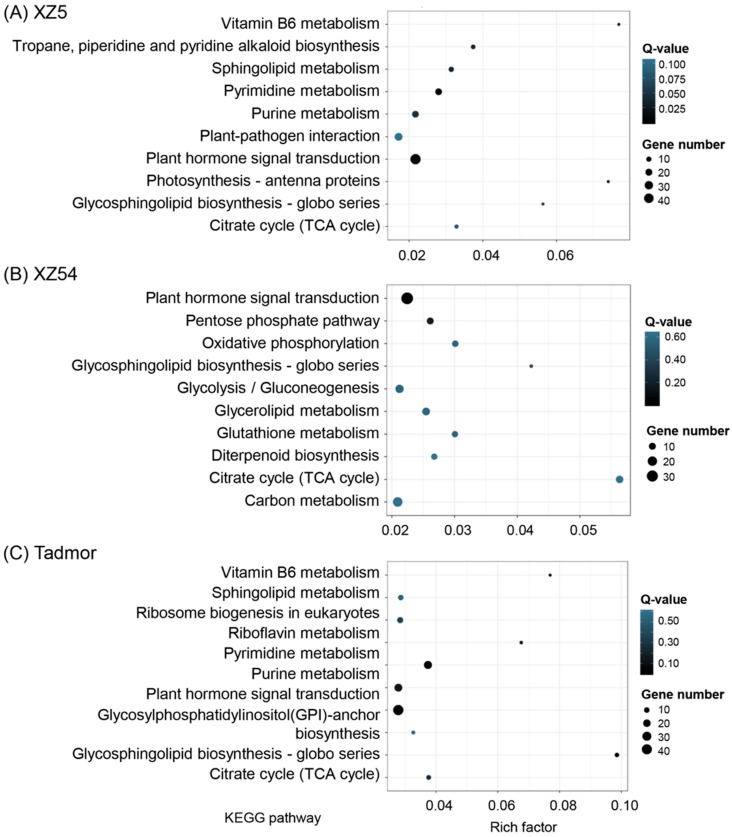
Kyoto Encyclopedia of Genes and Genomes (KEGG) enrichment analysis of differently expressed targets. KEGG pathway classification of target genes of differentially expressed miRNAs under drought stress in XZ5 (**A**), XZ54 (**B**) and Tadmor (**C**), respectively.

Putative targets were predicted for the 146 differently expressed miRNAs (31 conserved and 115 novel miRNAs) by the psRobot and TargetFinder. After filtering, a total of 2802 unique putative target genes were obtained (Table S7). Gene Ontology (GO) characterization were analyzed for screening target genes of differentially expressed miRNAs, which are mainly involved in cellular process, metabolic process, cell, cell part, organelle, binding and catalytic activity (Figure 7 and Table S8). The pathway enrichment of the differently expressed miRNA target genes was also performed based on the Kyoto Encyclopedia of Genes and Genomes (KEGG) database to gain further understanding of their biological functions (Figure 8 and Table S9). These target genes play roles mainly in transport, catabolism, signal transduction, transcription, translation, carbohydrate metabolism, amino acid metabolism and environmental adaptation, which demonstrated that the expressions of the miRNAs were induced or repressed to change the expression levels of their target genes to adapt to drought stress. Interestingly, the functions of these target genes of miRNAs were enriched on environmental adaptation, nucleotide metabolism, lipid metabolism and biosynthesis of other secondary metabolites in XZ5; while enriched on carbohydrate metabolism, signal transduction, energy metabolism and lipid metabolism in XZ54; whereas enriched on nucleotide metabolism, glycan biosynthesis and metabolism, signaling transduction and metabolism of cofactors and vitamins in Tadmor. These results suggested that different expression profiles of miRNAs and their target genes contributed to diverse tolerance ability against drought stress in three barley genotypes.

## 3. Discussion

**Figure 9:**
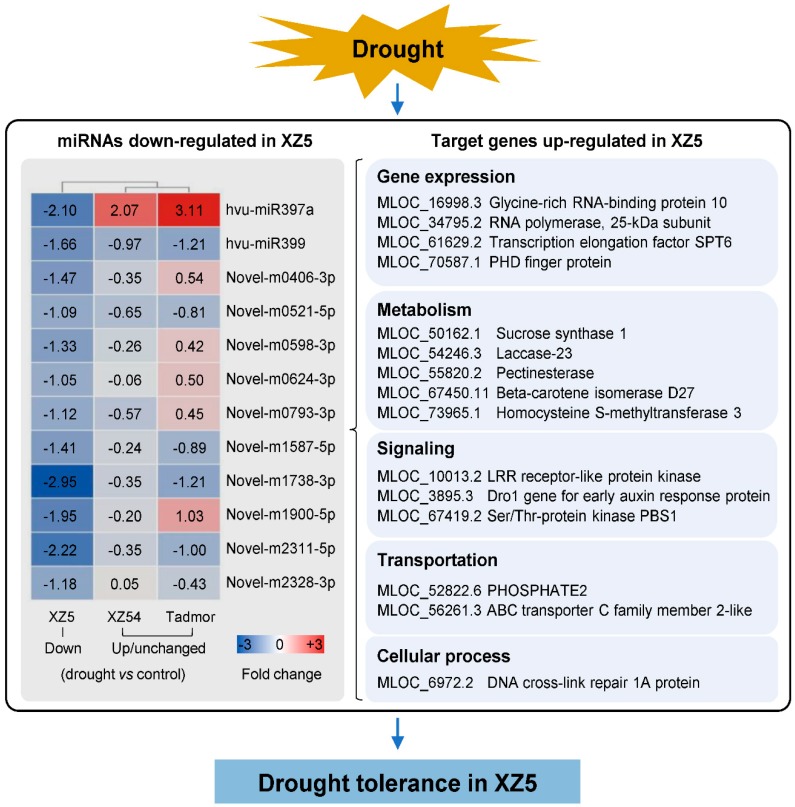
Hypothetical regulatory model involving miRNA and their target genes in drought tolerance of XZ5. Numbers in the heatmap represent the fold change of each miRNA in response to drought stress. The fold change (drought vs control) is log_2_N, log_2_N ≥ 1 are up-regulated, between 0 < |log_2_N| < 1 are unchanged and log_2_N ≤ −1 are down-regulated, *p*-values ≤ 0.01 and Q-value ≤ 0.001.

Drought is a major environmental perturbation that affects plant growth and development throughout the world. Therefore, it is vital to search for solutions to improve drought tolerance of crops. In the current study, a comparative analysis was performed on the miRNA transcriptome in roots of three barley genotypes in response to drought stress to identify genotypic-specific drought tolerant mechanisms, candidate drought tolerance associated miRNAs and target genes in Tibetan wild barley. In total, 43 differentially expressed miRNAs were identified. Among them, only two conserved and 10 novel miRNAs appeared to contribute to drought tolerance in XZ5, and were the focus for further analysis (Figure 9).

Recent studies showed that miRNAs were mainly involved in hormone signaling, cell growth, osmotic adjustment, and antioxidant. The high homology of conserved miRNAs provided important foundations for our study, and the prediction of novel miRNAs facilitated us to deeply understand their roles in drought stress response. The decreased expression of drought associated miRNAs in XZ5 led to the up-regulation of their target genes, which altered the downstream gene expression, cell wall components, plant hormone metabolism, stress signal transduction and element transport. This resulted in the regulation of root system architecture, maintaining the homeostasis of internal reactive oxygen species and the protection of biological macromolecules, leading to higher drought tolerance than XZ54 and Tadmor.

### 3.1. Drought-Tolerance Associated miRNAs Involved in Gene Expression

Glycine-rich RNA-binding protein (GR-RBP) families are involved in RNA transcription, which can bind RNA and DNA sequences with a preference to single-stranded nucleic acids. Yang et al. [20] reported that heterologous expression of *Arabidopsis thaliana GR-RBP 2* or *7* could improve grain yield and recovery rates of transgenic rice under drought stress. Tan et al. [21] found that overexpression of the *Malus prunifolia GR-RBP-1* gene could influence stomatal closure and reactive oxygen species (ROS) accumulation, improving salt stress tolerance in *Arabidopsis*. In our study, the target gene of Novel-m1900-5p was annotated as Glycine-rich RNA-binding protein 10, the down-regulation of Novel-m1900-5p and up-regulation of target gene *GR-RBP 10* might have protected against oxidative stress and confer drought tolerance in XZ5.

Plant homeodomain (PHD)-finger proteins are regarded as key factors in the regulation of transcription and chromatin structure in eukaryotes. Sun et al. [22] reported that there were eight members of the PHD-finger family up-regulated and three members down-regulated in rice in response to water deficits. Wang et al. [23] found that 17 *ZmPHD* genes were up-regulated by PEG treatment and six *ZmPHD* genes were strongly induced by ABA treatment in *Zea mays*. In our study, the PHD-finger proteins were predicted as the target gene of Novel-m0406-3p, which indicates they may participate in drought stress responses through ABA-dependent pathways in barley.

Suppressor of Ty (SPT) 6 is a conserved transcription regulator, which is viewed as an essential histone chaperone that mediates nucleosome reassembly during gene transcription. The SPT6-RNA polymerase II association plays a crucial role in controlling mRNA stability during transcription [24]. We found that the RNA polymerase (25-kDa subunit) and transcription elongation factor SPT6 were predicted as the target gene of Novel-m0598-3p and Novel-m2311-5p, respectively. Reduced expression of the two miRNAs together with increased expression of target gene in XZ5 may improve the stability of key mRNAs under drought stress, which contribute to the drought tolerance of XZ5.

### 3.2. Drought-Tolerance Associated miRNAs Involved in Metabolism

Sucrose is a dominant sugar transported to the sink organs of a plant, which plays an essential role in plant root development by releasing various signal molecules and interacting with plant hormones. Sucrose synthase catalyzes the reaction of converting sucrose into fructose and UDP-glucose, which has been thought to play a major role in sucrose metabolism. Jiang et al. [25] found, via proteome analysis, that sucrose synthase was up-regulated in wheat under water deficit conditions. Here, we identified sucrose synthase 1 as one of target genes of Novel-m0521-5p. The up-regulation of sucrose synthase 1 under drought conditions might have altered the root/shoot ratio and cell wall component, thereby enhancing the osmotic adjustment ability of XZ5.

Laccases (LAC), a blue copper-containing oxidases, catalyze the reaction of oxidizing monolignols to synthesize higher-order lignin, which are also regarded as participating in plant development and stress responses. Cai et al. [26] reported that root growth of a *lac* mutant in *Arabidopsis* was reduced under water stress. Cho et al. [27] reported that overexpression of a putative laccase precursor *OsChI1* gene increased drought tolerance in transgenic *Arabidopsis*. Zhao et al. [28] reported that the *LAC* was significantly down-regulated in the miR397a-overexpressed tomato with lower content of peroxidase (POD), superoxide dismutase (SOD) and polyphenol oxidase (PPO). As conserved miRNA, the laccases are also the target gene of miR397 in barley. In our study, hvu-miR397a was significantly down-regulated in XZ5, while up-regulated in XZ54 and Tadmor, so we hypothesized that hvu-miR397a is strongly related to drought tolerance in XZ5 as a regulator of lignin and antioxidant production.

Pectinesterase is a cell wall-associated protein involved in the synthesis of pectin, which supports plant adaptation to drought stress by modifying the root structure. Here, we identified a pectinesterase gene as the target gene of Novel-m0624-3p, which, together with sucrose synthase and the laccases mentioned above, may participate in cell wall and root system reconstruction under drought stress in XZ5, thus enhancing tolerance of water deficits.

Homocysteine S-Methyltransferase (HMT) 3, the target gene of Novel-m0521-5p in this study, encodes an enzyme that catalyzes the recycling of methionine in plants. As a basic amino acid, methionine directly affects the initiation of gene translation and protein synthesis, and indirectly participates in many physiological and biochemical pathways through downstream metabolites, especially the ethylene. Ogawa and Mitsuya [29] found that methionine metabolism-related genes such as *HMT* were induced by salt stress, which improved the resistance of *Arabidopsis* seeds and seedlings to salt stress. Szypulska et al. [30] identified a HMT protein induced by ABA and salt stress in cultivated barley by proteomic analysis. Beta-carotene isomerase D27, another target gene of Novel-m0521-5p, is the first enzyme involved in strigolactones (SLs) biosynthesis. As a new plant hormone, SLs has been reported as playing an essential role in plant growth regulation in various species under different abiotic stresses [31]. Thus, we speculate that the down-regulation of Novel-m0521-5p may induce the plant hormone ethylene and SLs to further regulate the growth and development of XZ5 in adapt to drought stress.

### 3.3. Drought-Tolerance Associated miRNAs Involved in Signaling

DEEPER ROOTING 1 (*DRO1*) is an early-auxin-response gene that is directly regulated by auxin response factors (ARFs) in the auxin signaling transduction. The expression of DRO1 is negatively regulated by auxin and is involved in auxin redistribution in the root tip [32]. Uga et al. [33] reported that DRO1 could alter the root system architecture to increase the deep rooting and maintain high yield performance under drought condition in a DRO1-introduced shallow-rooting rice cultivar. In our study, the Novel-m1738-3p strongly down-regulated in XZ5 comparing with XZ54 and Tadmor under drought stress. Therefore, we hypothesized that the target gene *DRO1* may be involved in root architecture modification in XZ5 in response to drought.

Leucine-rich repeat receptor-like kinase (*LRK*), which belongs to the largest subfamily of kinases in plants, plays important and diverse roles in abiotic stress responses [34]. Kang et al. [35] found that overexpression of the leucine-rich receptor-like kinase gene *LRK2* increases drought tolerance and tiller number in rice. Serine/threonine protein kinase AvrPphB susceptible 1 (PBS1) belongs to a subfamily of receptor-like cytoplasmic kinases. However, this kinase has been generally viewed as an important factor for plant innate immunity. *LRK* and *PBS1* were both predicted as the target gene of Novel-m0521-5p in this study, and may act as key factors in drought stress signaling.

### 3.4. Drought-Tolerance Associated miRNAs Involved in Transportation

miR399, a conserved miRNA, suppresses the expression of PHOSPHATE2 (*PHO2*) across many plant species. In our study, PHO2 was the target gene of both hvu-miR399 and Novel-m0793-3p in barley, PHO2 plays crucial roles in modulating phosphate transport. Baek et al. [36] found that miR399f played an important role in plant responses to abiotic stress, and the overexpression of miR399f resulted in a hypersensitive response to drought in transgenic *Arabidopsis*. Thus, the hyposensitive response to drought of XZ5 may be due to the down-regulation of hvu-miR399.

The ATP-binding cassette transporter C family (ABCC), a large and conserved family with transporter activity, play important roles in the export or import of various substrates across membranes. Park et al. [37] reported that the phytochelatin transporters *At*ABCC1 and *At*ABCC2 were important transporters for vacuolar sequestration, which play crucial roles in heavy metal stress. Here, we identified an ABCC2-like gene targeted by Novel-m1587-5p. As a phytochelatin transporter, it may influence the translocation of glutathione in XZ5, thus affect the drought tolerance.

## 4. Materials and Methods

### 4.1. Plant Materials and Drought Treatment

The greenhouse hydroponic experiment was performed at Zijingang Campus of Zhejiang University, Hangzhou, China, as described by Wu et al. [38] and Zhang et al. [39]. Two contrasting Tibetan wild barley genotypes XZ5 (drought-tolerant) and XZ54 (drought-sensitive) (*H. vulgare* L. ssp. *spontaneum*) identified in our previous screening by Zhao et al. [19], and a drought-tolerant *cv*. Tadmor [40,41] were used in this study. The seeds of XZ5, XZ54 and Tadmor were obtained from Huazhong Agricultural University and Zhejiang University, China, respectively. Healthy barley seeds of XZ5, XZ54 and Tadmor were germinated on sterilized moist sand and incubated at 20 °C /18 °C (day/night) in dark. Five-day-old uniform barley seedlings were transplanted to the lid of plastic containers, which filled with basic nutrient solution (BNS). The composition of BNS was described by Wu et al. [38], and solution was renewed every three days. Three days after transplanting, the seedlings in the control group and the drought group were treated with BNS and BNS + 20% PEG-6000, respectively. The experiment was arranged in a split-plot design with drought treatment as the main plot with 4 replicates. Root samples were harvested 24 h after drought treatment, and immediately frozen in liquid nitrogen, then stored at −80 °C for following experiments.

### 4.2. Small RNAs Library Construction and Next-Generation Sequencing

The total RNA samples in barley roots was extracted using the TRIzol reagent (Invitrogen, Carlsbad, CA, USA), and were further analyzed on 15% (*w*/*v*) denaturing polyacrylamide gel electrophoresis (PAGE), and RNA bands of 18 to 30 nucleotides were recovered and purified. Then the 3’ ends of the selected small RNA molecules were ligated to 5’-adenylated and 3’-blocked single-stranded DNA adaptors. Next, the reverse transcription (RT) primer was added to the system to cross with 3’ adaptors. After that, 5’ adaptors were linked to 5’ end of the products. The cDNA generated by RT-PCR with 100–120 bp in length were separated by PAGE gel to eliminate the primer-dimer and other byproducts. Finally, the sequencing process was performed by using BGISEQ-500 technology following the manufacturer’s instructions. [42,43].

### 4.3. Bioinformatics Analysis of Small RNAs

The clean small RNA reads were obtained after the removal of adaptor impurities and low-quality reads, and then were further compared with non-coding RNA sequences in the Rfam [44] and GenBank database [45]. Except for tRNA, rRNA, snRNA and snoRNA, the remaining sequences were compared with the reference genome of barley using Bowtie2 program [46]. Finally, they were subjected to a BLASTn search against miRBase database [47] to identify conserved miRNAs. Remaining unannotated clean small RNA sequences that perfectly matched with barley genome were subjected to RNAfold [48] and miReap to identify novel miRNAs by predicting precursors and the secondary structures. Those with typical stem-loop structure were regarded as novel miRNAs.

### 4.4. Differential Expression Profiles of miRNAs in Response to Drought Stress

The expression levels of miRNAs were calculated by using Transcripts Per Million (TPM) to eliminate the influence of sequencing discrepancy [49]. The DEGseq method based on the MA-plot was used to detect the differently expressed miRNAs [50]. Under the condition of *p*-values ≤ 0.01 and Q-value ≤ 0.001, we defined that the miRNAs with fold-changes > 1 were up-regulated, fold-changes within −1 to 1 were unchanged and fold-changes < −1 were down-regulated, respectively.

### 4.5. Target Gene Prediction and Functional Analysis

The putative target genes of conserved and novel miRNAs were predicted using psRobot [51] and TargetFinder [52] with default parameters. The expression profiles of these target genes were determined in our previous study [53]. In addition, all predicted target genes of differently expressed miRNAs were mapped to Gene Ontology (GO) terms using the Blast2GO pipeline [54]. Moreover, a Kyoto Encyclopedia of Genes and Genomes (KEGG) pathway enrichment analysis was performed to identify the involving pathways of differentially expressed miRNA target genes [55].

### 4.6. qRT-PCR Validation

Total RNA samples were also used for qRT-PCR validation. The quantity and quality of RNA was assessed using a Nanodrop ND-1000 (Nanodrop technologies, Houston, TX, USA). The first-strand cDNA was generated by Mir-X™ miRNA First-Strand Synthesis Kit (Takara, Japan) according to its instructions. The procedures of qRT-PCR were performed on LightCycler 480 System (Roche, Mannheim, Germany) using a TB Green Premix Ex Taq Kit (Takara, Japan), and 18s rRNA (Forward: GTGACGGGTGACGGAGAATT; Reverse: GACACTAATGCGCCCGGTAT) was used as the internal reference. The relative expression level of each miRNA was calculated using the comparative Ct method. Reverse transcription process was performed by polyadenylation using specific miRNA forward primer and mRQ 3’ primer provided in the kit used for qRT-PCR (Table S10).

## 5. Conclusions

The current study is the first to perform a comparative analysis of the miRNA transcriptome in roots of two Tibetan wild barley genotypes differing in drought tolerance and one drought tolerant cultivar in response to PEG treatment via next-generation sequencing. The expression profiles of seven conserved and 36 novel miRNAs were significantly different in response to drought stress among these barley genotypes. Further, two conserved and 10 novel miRNAs of these differentially expressed miRNAs were identified as drought-tolerance associated miRNAs in XZ5, which were mainly involved in gene expression, metabolism, signaling and transportation. These results preliminarily revealed the miRNA regulation mechanism in wild barley under drought stress, and also provided valuable candidate miRNAs and target genes for further investigation to alleviate drought stress in crops.

## Figures and Tables

**Table 1:** Summary of high-throughput sequencing of small RNAs from barley roots.

Library Type	XZ5 Control	XZ5 Drought	XZ54 Control	XZ54 Drought	Tadmor Control	Tadmor Drought
Total raw reads	27,450,467	26,932,023	27,644,000	27,800,513	28,585,892	28,370,268
Total clean reads	24,339,157 (88.67%)	24,392,408 (90.57%)	24,939,692 (90.22%)	25,771,155 (92.70%)	26,245,145 (91.81%)	25,550,026 (90.06%)
Mapped to Genome	17,310,505 (71.12%)	16,809,346 (68.91%)	18,034,873 (72.31%)	191,790,27 (74.42%)	18,772,380 (71.53%)	18,558,569 (72.64%)
Total miRNA reads	1,655,750 (6.80%)	1,401,558 (5.75%)	1,705,665 (6.84%)	1,312,933 (5.09%)	2,127,598 (8.11%)	1,553,489 (6.08%)
Conserved miRNAs	64	64	65	62	64	64
Novel miRNAs	944	797	972	877	1011	965

XZ5-Control, XZ54-Control and Tadmor-Control correspond to XZ5, XZ54 and Tadmor grown in BNS; XZ5-Drought, XZ54-Drought and Tadmor-Drought correspond to XZ5, XZ54 and Tadmor grown in BNS with 20% PEG, respectively.

**Table 2:** MicroRNAs associated with drought tolerance in Tibetan wild barley XZ5.

miRNA Name	Sequence	Fold Change			Target Gene	Annotation
		XZ5	XZ54	Tadmor		
hvu-miR397a	CCGUUGAGUGCAGCGUUGAUG	−2.10	2.07	3.11	*MLOC_54246.3*	Laccase-23 (*Oryza sativa*)
hvu-miR399	UGCCAAAGGAGAUUUGCCCCG	−1.66	−0.97	−1.21	*MLOC_52822.6*	PHOSPHATE2 (*Brachypodium distachyon*)
Novel-m0406-3p	CUUGGUCAAACUUAAAGAUGU	−1.47	−0.35	0.54	*MLOC_70587.1*	PHD finger protein (*Arabidopsis thaliana*)
Novel-m0521-5p	UCUUAUGUUGUGGGACGGAGG	−1.09	−0.65	−0.81	*MLOC_10013.2*	LRR receptor-like protein kinase (*A. thaliana*)
					*MLOC_50162.1*	Sucrose synthase 1 (*O. sativa*)
					*MLOC_67419.2*	Ser/Thr-protein kinase PBS1 (*A. thaliana*)
					*MLOC_67450.11*	Beta-carotene isomerase D27 (*O. sativa*)
					*MLOC_73965.1*	Homocysteine S-methyltransferase 3 (*B. distachyon*)
Novel-m0598-3p	UUUCGAUGUGCAACCAUGGUC	−1.33	−0.26	0.42	*MLOC_34795.2*	RNA polymerase, 25-kDa subunit (*A. thaliana*)
Novel-m0624-3p	UGAGCCGAACCAAUAUCACUC	−1.05	−0.06	0.50	*MLOC_55820.2*	Pectinesterase (*A. thaliana*)
Novel-m0793-3p	UGCCAAAGGAGAACUGCCCUG	−1.12	−0.57	0.45	*MLOC_52822.6*	PHOSPHATE2 (*B. distachyon*)
Novel-m1587-5p	UUGAAGAUUAGGUGGCGGCG	−1.41	−0.24	−0.89	*MLOC_56261.3*	ABC transporter C family member 2-like (*B. distachyon*)
Novel-m1738-3p	UGAACAUCCCAGAGCCACCGG	−2.95	−0.35	−1.21	*MLOC_3895.3*	Dro1 gene for early auxin response protein (*O. sativa*)
Novel-m1900-5p	UGCCUGGCUCCCUGUAUGCC	−1.95	−0.20	1.03	*MLOC_16998.3*	Glycine-rich RNA-binding protein 10 (*Brassica napus*)
Novel-m2311-5p	GGAAUGUUGUCUGGCUCGGG	−2.22	−0.35	−1.00	*MLOC_61629.2*	Transcription elongation factor SPT6 (*Vitis vinifera*)
Novel-m2328-3p	GCGUGCAAGGAGCCAAGCAUG	−1.18	0.05	−0.43	*MLOC_6972.2*	DNA cross-link repair 1A protein (*B. distachyon*)

Fold change (drought vs control) is log_2_N, log_2_N ≥ 1 are up-regulated, between 0 < |log_2_N| < 1 are unchanged and log_2_N ≤ −1 are down-regulated, *p*-values ≤ 0.01 and Q-value ≤ 0.001.

## Supplementary Materials

Supplementary materials can be found at https://www.mdpi.com/1422-0067/21/8/2795/s1.
